# Prognostic value of neutrophil-lymphocyte ratio, absolute lymphocyte count, and thrombocyte-lymphocyte ratio in predicting the outcomes of tetralogy of fallot primary repair

**DOI:** 10.3389/fcvm.2025.1489242

**Published:** 2025-06-05

**Authors:** Sisca Natalia Siagian, Christianto Christianto

**Affiliations:** ^1^ Pediatric Cardiology and Congenital Heart Defect Division, Department of Cardiology and Vascular Medicine, National Cardiovascular Center Harapan Kita, Universitas Indonesia, Jakarta, Indonesia; ^2^ Faculty of Medicine, Universitas Indonesia, Jakarta, Indonesia

**Keywords:** absolute lymphocyte count, complications, mortality, neutrophil-lymphocyte ratio, tetralogy of fallot, thrombocyte-lymphocyte ratio

## Abstract

Tetralogy of Fallot (ToF) is a chronic hypoxic condition that increases the risk of inflammatory responses during surgery. However, many inflammatory biomarkers used to predict surgical outcomes are costly and not widely accessible. This single-center, retrospective observational study aimed to compare the prognostic value of neutrophil-lymphocyte ratio (NLR), absolute lymphocyte count (ALC), and thrombocyte-lymphocyte ratio (TLR) in predicting outcomes of ToF primary repair. Between January 2020 and December 2022, 501 patients underwent ToF primary repair. Our findings revealed low postoperative reoperation (6.5%) and 30-day mortality (4.7%) rates, but prolonged LOS (92.2%) and a high complication rate (84%), with grade IVa (27.9%) and grade I (26.4%) complications being the most common. Elevated NLR (*r* = 0.132, *p* = 0.014), female gender (*r* = 0.117, *p* = 0.027), associated diagnoses (*r* = 0.104, *p* = 0.047), and prolonged surgical duration (*r* = 0.176, *p* = 0.001) were linked to higher complication burdens, though the correlations were weak. Among the three indexes, preoperative NLR demonstrated the strongest predictive value for complications, despite its low sensitivity and specificity. Given its accessibility and cost-effectiveness, NLR may serve as a useful tool for identifying high-risk patients and improving postoperative monitoring. Future multicenter prospective studies are necessary to validate the prognostic value of preoperative NLR under standardized conditions, across a broader patient population, and with more comprehensive confounding variables adjustments, ultimately improving ToF surgical outcomes (Clinical Trial NCT05976204).

## 1 Introduction

All surgical procedures, including congenital heart disease (CHD) surgeries, may induce inflammation as a result of a physiological response to trauma (1–3). In addition, most CHD operations are conducted at an early age and most of the organs are not fully developed, thus leading to poor prognosis and high risk of mortality (2). The use of cardiopulmonary bypass (CPB) may exacerbate this response and cause pathological complications as well as detrimental effects in various organs thus increasing morbidity and mortality in the postoperative period (2, 4, 5).

In the case of tetralogy of Fallot (ToF), chronic hypoxemia creates an imbalance between pro-inflammatory and anti-inflammatory responses in the preoperative period. This imbalance causes lethal injury to the myocardium which is then exacerbated by surgical trauma, CPB, and other factors resulting in prolonged mechanical ventilation time, duration of internsive care unit (ICU) stay, and total duration of hospitalization (2, 3, 6–9). Therefore, clinical indicators are required to help predict the morbidity and mortality risk of ToF surgery.

Various studies have recently shown some inflammatory biomarkers which may be closely related to increased morbidity and mortality in hypoxemic patients compared to those in acyanotic patients, but they are not readily available and expensive (4, 7, 10–12). Complete blood count with differential count was an inexpensive, repeatable, and widely available test for children undergoing ToF primary repair. Neutrophils are the first responders to inflammatory signals which are released from the damaged cells and tissues (3, 6, 9). On the contrary, lymphocytes decrease in response to inflammation as shown by its close relation with general health status and chronic inflammation state (13). Thrombocytes are not only involved in hemostasis, thrombosis, and wound healing, but also have important roles in immune regulation and inflammatory response (14).

Previous studies have shown the role of preoperative neutrophil-lymphocyte ratio (NLR) (15–21) and absolute lymphocyte count (ALC) (22–24) in predicting the outcome of congenital heart surgery. However, there were very limited studies on these inflammatory biomarkers as predictive indexes of ToF surgery outcomes (13, 19, 21). Despite its established association with coronary artery disease (25–29) and potential roles in pediatric cardiac surgery (30, 31), there was no study on preoperative thrombocyte-lymphocyte ratio (TLR) and its predictive value of ToF surgery. Furthermore, no study had compared these three preoperative indexes for their roles in predicting outcomes of ToF surgery. Therefore, this study aims to compare the prognostic value of these inflammatory biomarkers in predicting ToF surgery outcomes.

## 2 Materials and methods

### 2.1 Study population

This was a retrospective observational study on ToF primary repair in a single tertiary national cardiovascular center (National Cardiovascular Center Harapan Kita) between January 2020 and December 2022. The inclusion criteria were all ToF patients with any other associated cardiac anomalies, who underwent ToF primary repair and had a complete blood cell count with differential count available preoperatively. The exclusion criteria were as follows: surgery other than ToF primary repair, association with other procedures (except patent ductus arteriosus/PDA ligation, patent foramen ovale/PFO or atrial septal defect/ASD closure, or pulmonary arteries enlargement), preoperative hemodynamic instability, suspected or confirmed infection with prior antibiotic administration during the same hospital admission, and absence of complete blood count with differential count.

### 2.2 Preoperative and intraoperative data

All the data were obtained from the data registry in our center. The preoperative demographic data included were patients' gender, age, weight, oxygen saturation, and associated diagnoses other than ToF. The preoperative data of complete blood count and differential count must be tested from the most recent peripheral blood samples taken no later than 7 days before the surgery. The data obtained were the number of days of the most recent blood test including the leukocyte count, percentage neutrophil, percentage lymphocyte, and thrombocyte count, as well as the derived variables such as NLR, ALC, and TLR. The intraoperative data included were CPB time, aortic cross-clamp (AOX) time, and the total duration of surgery.

### 2.3 Evaluation and follow-up

Patients were evaluated and followed up for any complications and postoperative mortality during the same hospital stay until discharge. Patients were then followed up for any cause of mortality within 30 days postoperative. The primary endpoints were reoperation within the same hospital admission, mortality within 30 days postoperative, and complications. The complications were graded in severity using the modified Clavien-Dindo Complications Classification (CDCC) from least to most severe (grade I–V) (32). The Comprehensive Complication Index (CCI) was then used to convert these complications into cumulative severity scores using the formula $\sqrt{\sum \mathrm{Weigh}t_{\mathrm{comp}}}÷2$ (33). The secondary endpoints were hospital length of stay and postoperative length of stay.

### 2.4 Definition of variables

**NLR** was defined as the ratio of the absolute count of neutrophils to lymphocytes.

**TLR** was defined as the ratio of the absolute count of thrombocytes to lymphocytes.

**Reoperation** was defined as additional or corrective surgery after the initial primary ToF repair within the same hospital admission.

**Hospital length of stay (HLOS)** was defined as the duration of in-hospital stay from the initial admission to discharge.

**Longer postoperative LOS** was institutionally defined as in-hospital stay more than 5 days from perioperative to discharge.

**Complication** was defined as any adverse events that arose perioperative or postoperative to discharge.

### 2.5 Statistical analysis

Categorical data were expressed as counts and percentages. Kolmogorov–Smirnov test was used as a normality test for continuous variables expressed as means ± standard deviations in normally distributed data or median and interquartile range (IQR), in not normally distributed data. Student *T* test (normal distribution) or Mann–Whitney test (not normal distribution) was used to compare two groups with numerical data. Anova test (normal distribution) or Kruskal–Wallis test (not normal distribution) was used to compare more than two groups with numerical data. Chi-square test and Fischer's exact test (if did not meet the criteria for Chi-square test) were used for categorical data due to the small sample size. Pearson test (normal distribution) and Spearman test (not normal distribution) were used for correlation tests. In general, *p*-value <0.05 was considered statistically significant. Univariate and multivariate analyses were performed to evaluate the association between preoperative indexes with significant outcomes, based on the univariate analysis. Receiving Operating Characteristic (ROC) curve analysis was used to determine the optimal cutoff levels of the preoperative NLR, ALC, and TLR as well as comparing these markers to predict the significant outcomes. The statistical analyses were performed using SPSS 26.0.

### 2.6 Ethical approval

This study has been reviewed and approved by the Research Ethics Committee of National Cardiovascular Center Harapan Kita. No informed consent was needed from the patients as this study used secondary data from the database.

## 3 Results

### 3.1 Baseline characteristics and outcomes

Based on the inclusion and exclusion criteria, a total of 387 patients who underwent ToF primary repair between January 2020 and December 2022 were retrospectively enrolled. Most of the patients were male (56.6%), had associated diagnoses other than ToF (63.8%), and weighed ≥10 kg (64.6%). Other preoperative data of the patients are summarized in Table 1. The most common associated diagnosis was PDA (28.7%), followed by ASD (27.9%) with ASD secundum as the most common subtype (15.8%), see Table 2.

**Table 1:** Preoperative data of the included patients.

Variable	Groups	*N* (%)/Median (IQR)/Mean (SD)	95% CI (%)
Age (months)		44.8 (21.8–99.8)	69.1–86.1
Gender	Female	168 (43.4)	
	Male	219 (56.6)	
Weight (kg)		12 (8.5–18)	15.8–18.6
	<10 kg	137 (35.4)	
	≥10 kg	250 (64.6)	
SpO_2_ (%)		77 (69–86)	75–77.7
Associated Diagnosis	No	140 (36.2)	
	Yes	247 (63.8)	
Preoperative Days Blood Obtained		3 (2–5)	3.2–3.6
Leukocyte Count		8,570 (7,190–10,150)	8,719.7–9,239.7
Neutrophil Percentage		42.1 (14.7)	40.6–43.5
Lymphocyte Percentage		46.5 (14.3)	45–47.9
Thrombocyte Count		2,99,685.7 (1,14,658)	2,88,226.3–3,11,145.1
NLR		0.88 (0.56–1.43)	1.06–1.4
ALC		3,854 (2,806–5,299)	3,994.6–4,374.7
TLR		72.3 (54.89–98.9)	79.09–88.49

ALC, absolute lymphocyte count; CI, confidence interval; IQR, interquartile range; NLR, neutrophil lymphocyte ratio; SpO_2_, blood oxygen saturation measured by pulse oximetry; TLR, thrombocyte lymphocyte ratio; kg, kilogram.

**Table 2:** Associated diagnosis of the patients.

Associated Diagnosis		*N*	%
Down Syndrome		7	2
Persistent Left SVC		10	2.6
Persistent Left IVC		1	0.3
ASD		108	27.9
	Sinus Venosus	1	0.3
	Secundum	61	15.8
	PFO	46	11.9
VSD Muscular		1	0.3
VSD outlet		5	1.3
AVSD incomplete		2	0.5
PDA		111	28.7
MAPCAs		45	11.6
PA Stenosis		15	3.9
PA Hypoplasia		6	1.6
PV Stenosis		2	0.5
PV Atresia		32	8.3
PV Absent		5	1.3
Others		3	0.8
	PA from PDA	1	0.3
	Coronary AV fistula	1	0.3
	Dextrocardia	1	0.3

ASD, atrial septal defect; AV, arteriovenous; AVSD, atrioventricular septal defect; IVC, inferior vena cava; MAPCAs, major aortopulmonary collateral arteries; PA, pulmonary artery; PDA, patent ductus arteriosus; PFO, patent foramen ovale; PV, pulmonary valve; SVC, superior vena cava; VSD, ventricular septal defect.

The median of CPB time, AOX time, and surgery duration were 103 (82–128) min, 53 (42–67) min, and 241 (210–300) min respectively, see Table 3 for more details. There were 25 patients (6.5%) who needed reoperation due to residual ventricular septal defect and residual pulmonary stenosis as well as 18 deaths (4.7%) due to complications. The median hospital length of stay (HLOS) was 9 (6–14) days and the postoperative LOS was 7 (5–10) days. Most of the patients had complications (84%) with majority of grade IVa complications (27.9%), followed by grade I complications (26.4%). The median CCI was 25.7 (8.7–44.3), see Table 4. The recorded complications were cardiac complication (arrhythmia, pericardial effusion or tamponade, low cardiac output syndrome or right heart failure, ventricular dysfunction, cardiac arrest), pulmonary complication (pneumonia, pleural effusion, hemothorax, pneumothorax, atelectasis), kidney complication (acute kidney injury), other complications (anemia, thrombocytopenia, metabolic acidosis, electrolyte imbalance), including death.

**Table 3:** Intraoperative data of the patients.

Variable	Groups	N (%)/Median (IQR)	95% CI (%)
CPB time (min)		103 (82–128)	106.6–123.4
	<100 min	186 (48.1)	
	≥100 min	201 (51.9)	
AOX time (min)		53 (42–67)	55.6–61.1
Surgery duration (min)		241 (210–300)	252.1–271

AOX, aortic cross clamp; CI, confidence interval; CPB, cardiopulmonary bypass; IQR, interquartile range.

**Table 4:** Outcomes of the patients.

Variable	Groups	*N* (%)	95% CI (%)
Reoperation	No	362 (93.5)	
	Yes	25 (6.5)	
Mortality	No	369 (95.3)	
	Yes	18 (4.7)	
HLOS (days)		9 (6–14)	10.9–12.6
Postoperative LOS (days)		7 (5–10)	8.7–10.1
	<5 days	30 (7.8)	
	≥5 days	357 (92.2)	
Complications	No	62 (16)	
	Yes	325 (84)	
Grade of Complication	No	62 (16)	
	I	102 (26.4)	
	II	31 (8)	
	III	54 (14)	
	IIIa	24 (6.2)	
	IIIb	30 (7.8)	
	IV	120 (31)	
	IVa	108 (27.9)	
	IVb	12 (3.1)	
	V	18 (4.7)	
CCI		25.7 (8.7–44.3)	28.1–33.4

CCI, comprehensive complication index; CI, confidence interval; CDCC, Clavien-Dindo complications classification; HLOS, hospital length of stay; LOS, length of stay.

### 3.2 Univariate analysis

Univariate analyses between preoperative NLR, ALC, and TLR with the patients' outcomes were summarized in Tables 5, 6.

**Table 5:** Univariate categorical analysis of ToF surgery outcomes.

Variables	Groups	NLR	*p*-value	ALC	*p*-value	TLR	*p*-value
Mortality	No	0.87 (0.56–1.39)	0.530	3,864 (2,802.5–5,282.5)	0.925	72.19 (54.82–98.84)	0.471
	Yes	0.9 (0.57–2.03)		3,632 (2,800.25–5,449.5)		81.82 (54.43–123.82)	
Reoperation	No	0.88 (0.57–1.37)	0.922	3,853.5 (2,807.5–5,258.5)	0.761	72.25 (55.21–98.55)	0.992
	Yes	0.88 (0.4–2.35)		4,218 (2,290.5–5,556.5)		73.69 (49.68–116.86)	
Complications	No	0.74 (0.47–1.26)	0.077	3,802 (2,640–5,497)	0.832	64.89 (53.42–89.15)	0.126
	Yes	0.89 (0.59–1.43)		3,879 (2,855–5,261)		76.37 (55.33–100.33)	
Grade of Complication	No	0.74 (0.47–1.26)	0.100	3,802 (2,640–5,497)	0.019*	64.89 (53.42–89.15)	0.274
	I	0.85 (0.55–1.32)		4,192.5 (3,348.8–5,752.5)		71.93 (54.43–96.14)	
	II	0.89 (0.66–1.69)		3,942 (2,808–5,266)		65.53 (42.14–97.6)	
	IIIa	1.11 (0.63–1.66)		3,616 (2,501.5–4,351.5)		87.32 (64.57–100.34)	
	IIIb	1.23 (0.71–1.86)		3,063 (2,172.5–4,128.3)		77.91 (61.96–116.83)	
	IVa	0.9 (0.55–1.35)		3,704.5 (2,577.5–5,160.8)		78.56 (53.99–104.82)	
	IVb	0.83 (0.53–0.98)		4,462.6 (3,270–5,186.3)		61.14 (37.26–115.29)	
	V	0.9 (0.57–2.03)		3,632 (2,800.3–5,449.5)		81.82 (54.43–123.82)	
Postoperative LOS	<5 days	0.92 (0.57–1.43)	0.822	3,732.5 (3,004–4,960.8)	0.746	87.95 (67.64–115.5)	0.026*
	≥5 days	0.87 (0.56–1.42)		3,882 (2,795–5,360.5)		71 (54.68–98.46)	

* Significant *p*-value (<0.05).

ALC, absolute lymphocyte count; NLR, neutrophil lymphocyte ratio; TLR, thrombocyte lymphocyte ratio.

**Table 6:** Univariate numerical analysis of ToF surgery outcomes.

Variables	Correlation coefficient (NLR)	*p*-value	Correlation coefficient (ALC)	*p*-value	Correlation coefficient (TLR)	*p*-value
HLOS	−0.008	0.879	−0.002	0.961	−0.161	0.001*
Postoperative LOS	−0.01	0.844	0.026	0.603	−0.157	0.002*
CCI	0.87	0.086	−0.078	0.126	0.095	0.061

* Significant *p*-value (<0.05).

ALC, absolute lymphocyte count; HLOS, hospital length of stay; LOS, length of stay; NLR, neutrophil lymphocyte ratio; TLR, thrombocyte lymphocyte ratio.

#### 3.2.1 Neutrophil-Lymphocyte ratio

Higher preoperative NLR was observed in patients with mortality and complications, though the association lacked statistical significance. Preoperative NLR showed a strong positive correlation with CCI, though not statistically significant. Conversely, higher preoperative NLR was observed in patients with shorter HLOS and postoperative LOS, but without statistical significance.

#### 3.2.2 Absolute lymphocyte count

Preoperative ALC was significantly associated with complication grade, though inconsistently. Median preoperative ALC was lower in non-survivors but higher in patients undergoing reoperation or experiencing complications. Weak, non-significant correlations were observed between preoperative ALC and HLOS, postoperative LOS, and CCI.

#### 3.2.3 Thrombocyte-Lymphocyte ratio

Higher preoperative TLR was linked to poorer outcomes (mortality, reoperation, complication), though not statistically significant. Preoperative TLR showed a weak positive correlation with CCI and weak negative correlations with HLOS and postoperative LOS, all without statistical significance. Conversely, higher preoperative TLR was associated significantly with postoperative LOS < 5 days.

### 3.3 Univariate and multivariate logistic regression analysis of complications

We conducted univariate analyses, then a multivariate logistic regression to determine the association between NLR, TLR, and ALC with complication of ToF surgery which was categorized into two groups (without complication and with complication). Based on the univariate analysis, variables included in the multivariate analysis were weight, associated diagnosis, NLR, and TLR. Even though lacking statistical significance, there was a higher risk of complication in patients with higher NLR and no associated diagnosis (Table 7).

**Table 7:** Univariate and multivariate logistic regression analysis of categorized complications.

Variables	Univariate		Multivariate	
	OR (95% CI)	*p*-value	Adjusted OR (95% CI)	*p*-value
Gender (Female)	1.16 (0.67–2.02)	0.592		
Age (months)	1 (1–1)	0.316		
Weight (kg)	1.01 (0.99–1.03)	0.117	1 (0.98–1.03)	0.840
Weight (<10 kg)	0.72 (0.41–1.25)	0.240		
SpO_2_ (%)	0.99 (0.97–1.02)	0.724		
Associated Diagnosis (Yes)	1.69 (0.98–2.93)	0.058	1.71 (0.98–2.96)	0.058
NLR	1.21 (0.87–1.68)	0.077	1.22 (0.79–1.86)	0.371
ALC	1 (1–1)	0.832		
TLR	1 (1–1.01)	0.126	1 (0.99–1.01)	0.879
Surgery Duration (minutes)	1 (1–1)	0.637		
CPB time (minutes)	1 (1–1.01)	0.654		
CPB (<100 min)	1.18 (0.69–2.04)	0.541		
AOX time (minutes)	1 (0.99–1.01)	0.532		

ALC, absolute lymphocyte count; CI, confidence interval; IQR, interquartile range; NLR, neutrophil lymphocyte ratio; SpO_2_, blood oxygen saturation measured by pulse oxymetry; TLR, thrombocyte lymphocyte ratio; kg, kilogram AOX, aortic cross clamp; CI, confidence interval; CPB, cardiopulmonary bypass; IQR, interquartile range.

### 3.4 ROC curve

**Figure 1:**
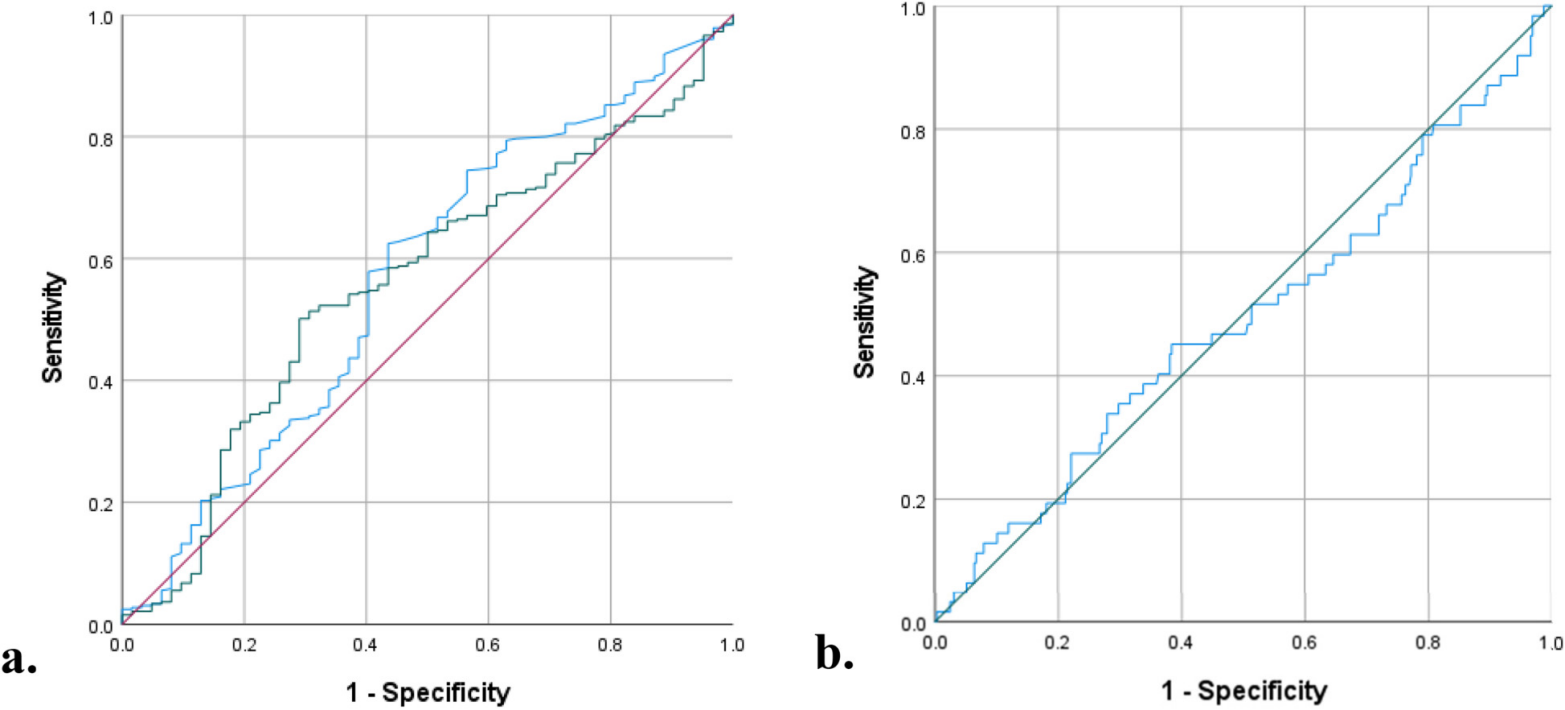
ROC curves: **(a)** preoperative NLR (blue curve) vs. TLR (green curve) **(b)** Preoperative ALC (blue curve), for prediction of complication.

**Figure 2:**
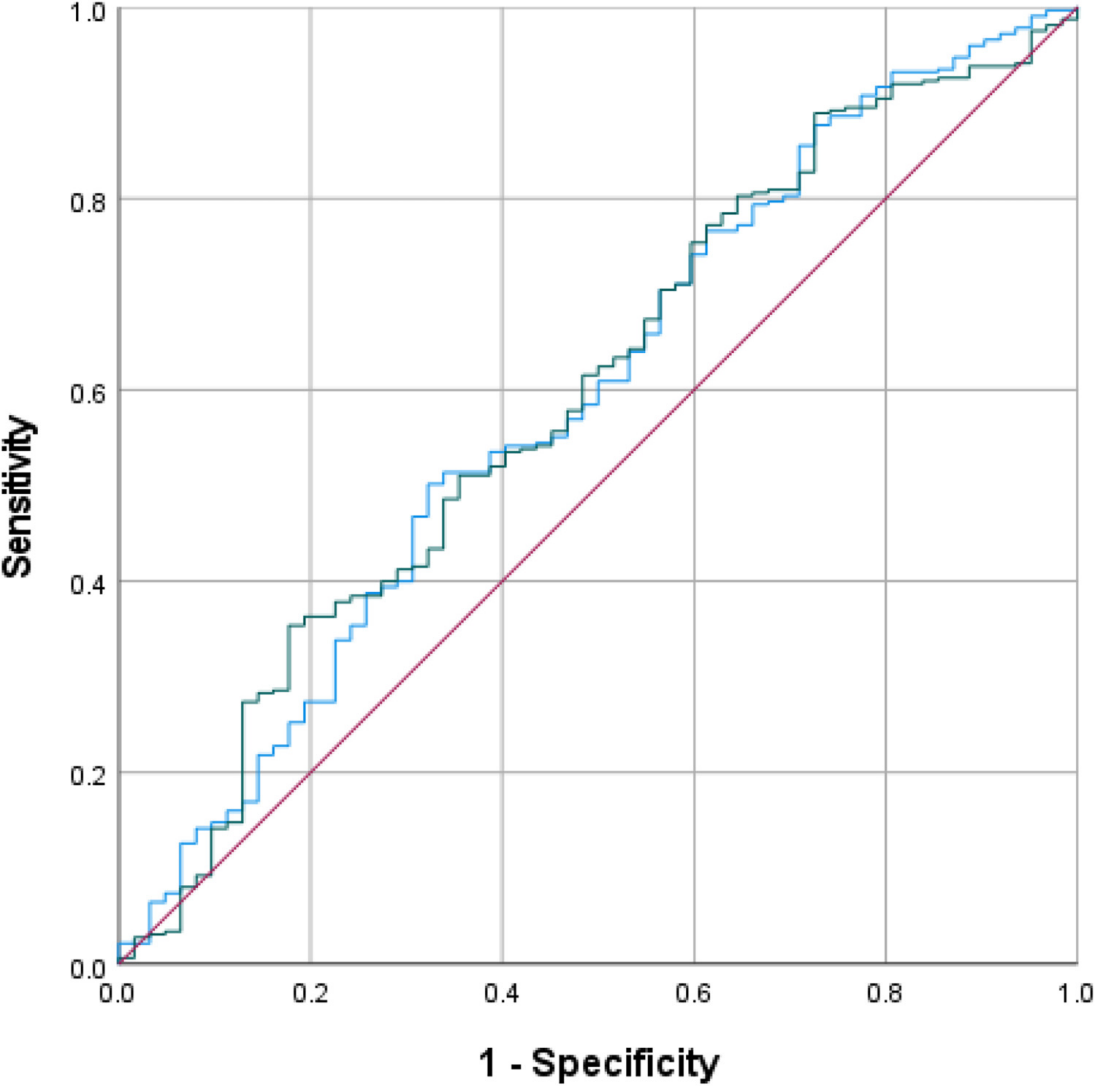
ROC curves for the model with (blue curve) and without (green curve) preoperative NLR.

First, both preoperative NLR, ALC, and TLR were compared using ROC curves for the value in predicting complication in ToF primary repair (Figure 1). The areas under the curve (AUC) were the highest in preoperative NLR (0.571, 95% CI: 0.490–0.652; *p* = 0.077), followed by TLR (0.561, 95% CI: 0.486–0.637), and ALC (0.492, 95% CI: 0.409–0.575). Based on the ROC curve analysis, the cutoff values for preoperative NLR, TLR, and ALC as a predictor of moderate and severe complications were >0.61 (sensitivity: 75%, specificity: 57%), >76 (sensitivity: 50%, specificity: 29%), and >4346 (sensitivity: 40%, specificity: 38%), respectively. Using the model from multivariate logistic regression analysis, we compared the ROC curves using 2 models: one model which included NLR and another model which excluded NLR. In the model with preoperative NLR, the AUC was 0.591 (95% CI: 0.512–0.671, *p* = 0.022). Another model without preoperative NLR showed similar AUC of 0.594 (95% CI: 0.516–0.673, *p* = 0.019), see Figure 2.

### 3.5 Univariate and multivariate linear regression analysis of CCI

**Figure 3:**
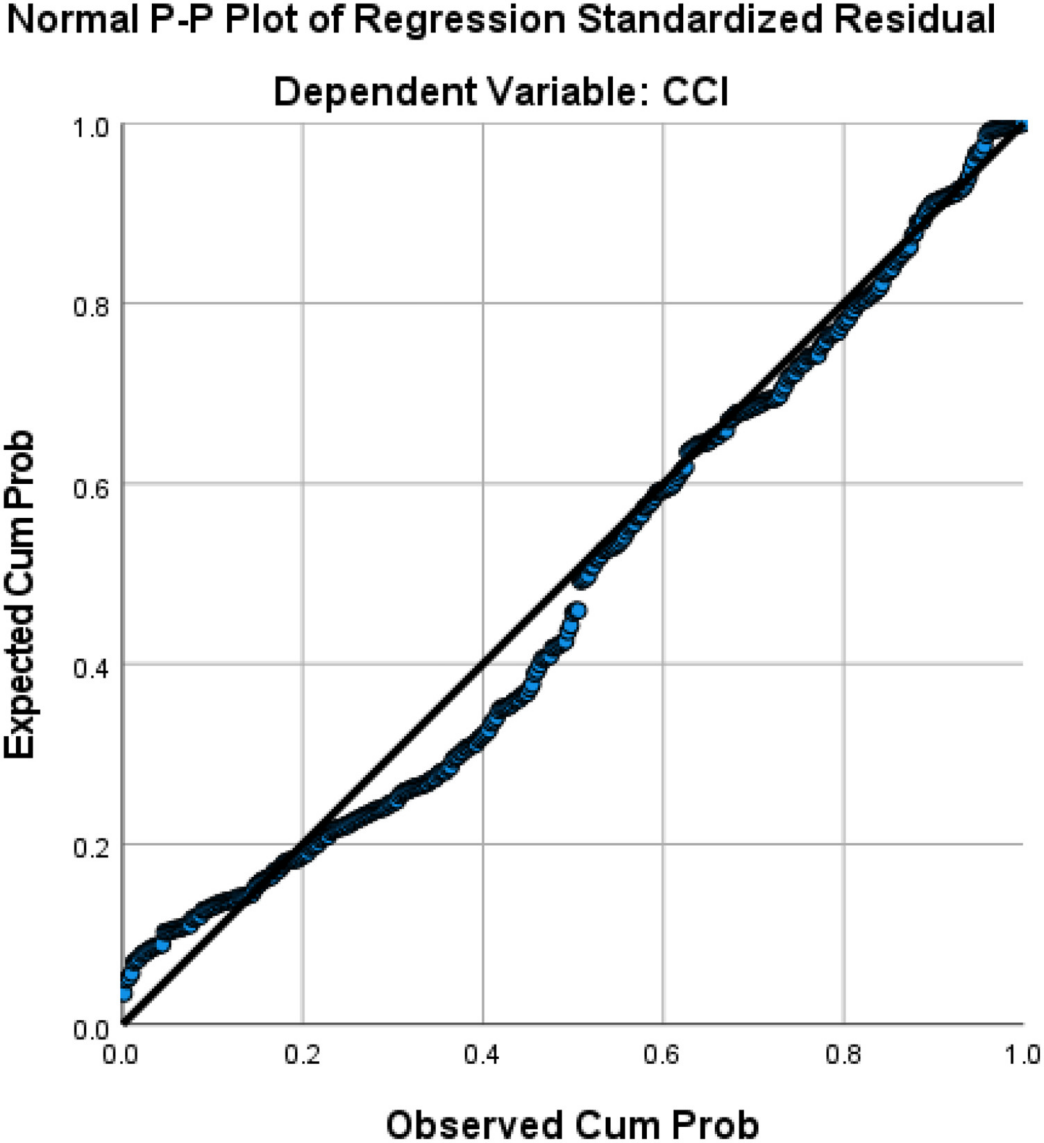
Regression linear plot of CCI.

Gender, associated diagnosis, NLR, ALC, TLR, surgery duration, CPB time, and AOX time were correlated significantly with CCI. However, multivariate linear regression revealed that only gender, associated diagnosis, NLR, and surgery duration were significantly correlated with CCI, though the correlations were weak (R 0.294, R^2^ 8.6%, Adjusted R^2^ 7.5%, Standard Error of the Estimate 25.3), see Table 8. Based on the regression **(**Figure 3), the following equation was produced: CCI = 7.901 + 6.186*Gender (Female = 1, Male = 0) + 5.754*Associated Diagnosis (Yes = 1, No = 0) + NLR*2.147 + Duration of Surgery*0.052.

**Table 8:** Univariate and multivariate linear regression analysis of comprehensive complication index.

Variables	Univariate		Multivariate	
	Coefficient correlation	*p*-value	Coefficient correlation	*p*-value
Gender (Female)	0.101	0.046*	0.117	0.027*
Age (months)	0.007	0.898		
Weight (kg)	0.028	0.585		
SpO_2_ (%)	−0.040	0.431		
Associated Diagnosis (Yes)	0.141	0.006*	0.104	0.047*
NLR	0.087	0.086*	0.132	0.014*
ALC	−0.078	0.126*		
TLR	0.095	0.061*		
Surgery Duration (minutes)	0.135	0.011*	0.176	0.001*
CPB time (minutes)	0.175	<0.001*		
AOX time (minutes)	0.124	0.017*		

* Significant *p*-value <0.05.

ALC, absolute lymphocyte count; CI, confidence interval; IQR, interquartile range; NLR, neutrophil lymphocyte ratio; SpO_2_, blood oxygen saturation measured by pulse oxymetry; TLR, thrombocyte lymphocyte ratio; kg, kilogram AOX, aortic cross clamp; CI, confidence interval; CPB, cardiopulmonary bypass; IQR, interquartile range.

## 4 Discussion

Tetralogy of Fallot (ToF) is a common cyanotic congenital heart diseases that leads to chronic hypoxia, disrupting long-term energy metabolism and causing myocardial and systemic organ injury (34, 35). This condition induces hypoxia-related inflammation due to an imbalance between pro-inflammatory and anti-inflammatory responses, increasing the expression of genes linked to apoptosis and remodeling while decreasing the expression of genes associated with myocardial contractility and function. Consequently, myocardial stress triggers the activation of various genes, including cytokines such as IL-6 and TNF-α, further contributing to inflammatory processes (8, 36, 37).

Since ToF often coexists with associated diagnoses, we did not exclude such cases to ensure our findings can also reflect these conditions. Additionally, we consider RVOT obstruction and shunting as the baseline physiology, with minimal impact from PDA, ASD, and similar anomalies (38). Our study found that most ToF primary repairs resulted in prolonged LOS (92.2%), likely due to preoperative inflammation increasing postoperative morbidity and mortality (2, 4, 5). Cyanotic CHD, including ToF, is an independent risk factor for exacerbated systemic inflammatory response syndrome (SIRS) after cardiopulmonary bypass (CPB) (3, 6, 7). Additional risks include young age, low weight, extended CPB and aortic cross-clamp times, preoperative leukocytosis, and postoperative liver dysfunction (3, 6). This inflammatory burden leads to prolonged ICU stays, mechanical ventilation, and hospitalization, posing challenges in ToF surgical management (2, 34).

Our study observed a low mortality rate of 4.7% following ToF primary repair, with only 6.5% requiring reoperation, consistent with previous findings of a 0%–2% surgical mortality rate, even in neonates (39–41). Despite its overall safety, ToF primary repair is frequently associated with ICU and postoperative morbidity, which can contribute to mortality (42). In our study, 84% of patients experienced complications either during or after surgery, with grade IVa complications (27.9%) being the most common, followed by grade I complications (26.4%). These findings highlight the importance of identifying patients at risk for postoperative morbidity and mortality based on their preoperative characteristics to improve outcomes.

There were many studies on potential inflammatory biomarkers for predicting increased morbidity and mortality in hypoxemic patients (4, 7, 10–12). However, these biomarkers are expensive and may not be readily available in some centers, especially those in developing countries. The three indexes NLR, ALC, and TLR which are derived from complete blood count with differential count are very attractive alternatives to these biomarkers as they may be obtained from a simple, conventional, inexpensive, repeatable, and widely available blood test for children undergoing ToF primary repair.

Our study found that patients with mortality and complications following ToF primary repair had higher preoperative NLR. Manuel et al. identified a link between preoperative NLR and prolonged ICU and hospital LOS and its potential role in predicting grade III acute kidney injury following ToF primary repair (19, 21). Conversely, higher preoperative NLR was linked to shorter HLOS and postoperative LOS, possibly due to the time gap between admission, blood specimen collection, and surgery. While our analysis showed a significant association between preoperative ALC and complication grade, the trend was inconsistent. This result was supported by finding by Wu et al. that low preoperative ALC may predict poor prognosis in children undergoing ToF repair (13). We observed weak negative correlations between preoperative TLR and both HLOS and postoperative LOS, though these appeared coincidental, with higher preoperative thrombocyte levels possibly offering protection against complications like bleeding.

In our study, preoperative NLR, ALC, and TLR were not significantly associated with complications in univariate and multivariate analysis. However, preoperative NLR emerged as the strongest predictor among the three with a cutoff of >0.61 (sensitivity: 75%, specificity: 57%, AUC = 0.571, *p* = 0.077). Previous studies by Manuel et al. reported higher cutoff values for predicting longer HLOS (>0.80) and grade III acute kidney injury (>0.93) (19, 21). Other cardiac surgeries have shown even greater preoperative NLR thresholds, such as >3.23 in general cardiac surgery, >3.4 in coronary artery bypass graft (CABG), and >8 in emergent surgery for acute type I aortic dissection to predict mortality (43–45). Despite its predictive potential, ROC curve analyses demonstrated that preoperative NLR was unreliable for predicting complications in ToF primary repair, possibly due to differences in study design and the types of complications analyzed. Nonetheless, previous studies have linked preoperative NLR with cardiovascular surgery complications including atrial fibrillation, acute kidney injury, and mortality (21, 44–46).

Conversely, preoperative NLR, ALC, and TLR were significantly correlated with complication severity based on comprehensive complication index (CCI). Multivariate analysis revealed that only preoperative NLR (*p* = 0.014) along with gender (*p* = 0.027), associated diagnosis (*p* = 0.047), and duration of surgery (*p* = 0.001) had significant correlations with CCI. Tasoglu et al. identified preoperative NLR as an independent predictor of saphenous vein graft patency in CABG patients. Their study categorized patients into three groups based on preoperative NLR levels (low, intermediate, and high), demonstrating that higher NLR was linked to an increased incidence of saphenous vein graft failure (47). This finding aligns with our results, where higher preoperative NLR correlated with higher CCI. However, the relatively weak correlation in our study may be attributed to methodoclical differences, particularly blood sample collection timing (up to 7 days in our study vs. 1–3 days in other studies) (21, 45, 47).

Our findings indicate that female ToF patients experienced greater postoperative complications. Women are generally at greater risk of morbidity and mortality in cardiac surgery, often presenting at an older age with more comorbidities and atypical symptoms, making them more likely to require urgent than elective revascularization (48, 49), Similarly, Kochilas et al. reported higher mortality rates among girls undergoing high-risk cardiac surgeries, reinforcing our findings (50). Chromosomal and gonadal hormone factors have been shown to play a role in sex-specific responses to cardiac ischemic or hypoxic injury (51–53). However, some studies, including a meta-analysis, have found no significant gender differences in pediatric cardiac surgery outcomes (54, 55).

Patients with additional diagnoses beyond ToF experienced higher cumulative complications following ToF repair in our study. Previous researches have shown that individuals with genetic disorders or other cardiac anomalies, such as coronary abnormalities, tend to have poorer surgical outcomes (56, 57). These patients often present with compromised baseline health compared to those with isolated ToF, which may contribute to increased postoperative risks. Furthermore, the presence of complex cardiac anatomy can make can make surgical repair more challenging, further elevating the likelihood of morbidity and mortality.

Our study found that longer surgery duration, CPB time, and AOX time were associated with a higher complication burden, though regression analysis identified surgery duration as the only statistically significant factor. Prolonged operative time, including extended CPB and AOX durations, is known to exacerbate inflammatory responses in cardiac surgery (3, 6). A systematic review and meta-analysis have linked longer surgical procedures to an increased risk of complications (58). The prolonged CPB and AOX times likely contribute to heightened inflammation through ischemia-reperfusion injury, further impacting postoperative outcomes (3, 6, 59).

Previous studies have shown that patients with oxygen saturation <90% exhibit elevated preoperative or intramyocardial cytokine levels (7, 36). Additionally, researches have linked increased IL-6 with high neutrophils counts and low lymphocytes levels in chronically hypoxia patients, indicating a potential inflammatory imbalance (8, 9). Chronic hypoxia may also contribute to reperfusion injury caused by oxygen-derived free radicals, leading to myocardial and endothelial damage and remodeling, similar to patients undergoing CABG (60). CABG. However, the lack of significant findings in our study may be attributed to the presence of associated cardiac anomalies, such as PDA, ASD, PFO, AVSD, and MAPCAs, which could have influenced the results.

Low body weight has traditionally been considered a risk factor for exacerbated inflammatory responses in cardiac surgery (3, 6). However, our study found that patients weighing less than 10 kg had a lower complication risk, suggesting that advancements in surgical techniques may have contributed to improved outcomes. A study on very low birth weight infants (<2.2 kg) also reported relatively favorable cardiac surgery results, despite an increased risk of ICU morbidity and mortality (61). Additionally, higher body weight was associated with older age and prolonged exposure to chronic hypoxia-induced inflammation, which may contribute to a poorer prognosis.

## 5 Limitations

As a single-center, retrospective study, our study is susceptible to recall bias and missing data. However, we minimized this risk by utilizing a comprehensive, updated database. The study population was heterogeneous, with multiple potential confounding factors. While we attempted to address bias through multivariate analysis, the scope of included variables was constrained by data limitations. Another limitation was the use of blood tests conducted up to seven days before surgery due to institutional regulations, which may not fully reflect the patients' preoperative condition. Additionally, CRP was not included as a standard preoperative laboratory investigation, limiting the assessment of inflammatory profiles. Lastly, patient outcome evaluations were restricted to 30 days, as many participants traveled from across the country and preferred to return to their hometowns post-surgery.

## 6 Conclusion

Elevated preoperative NLR was associated with increased complication burdens in ToF primary repair, as well as factors such as female gender, associated diagnoses, and prolonged surgical duration. Although the correlation was weak, with low sensitivity and specificity, preoperative NLR proved to be the most reliable among the three indexes for predicting complications. Its simplicity, cost-effectiveness, and accessibility make it a promising tool for identifying high-risk patients and optimizing postoperative monitoring. Future multicenter prospective studies are necessary to validate the prognostic value of preoperative NLR under standardized conditions, across a broader patient population, and with more comprehensive confounding variables adjustments, ultimately improving ToF surgical outcomes.

## Data Availability

The dataset associated with this study is not readily available due to strict institutional policies that govern data sharing and confidentiality. These policies ensure compliance with regulatory and ethical guidelines, particularly concerning sensitive information and proprietary data. Requests to access the dataset should be directed to the corresponding author and we would further decide whether to share the dataset based on the purposes.
